# Time Course of Heart Rate Variability Response to PM_2.5_ Exposure from Secondhand Smoke

**DOI:** 10.1371/journal.pone.0154783

**Published:** 2016-05-25

**Authors:** Jennifer L. Garza, Murray A. Mittleman, Jinming Zhang, David C. Christiani, Jennifer M Cavallari

**Affiliations:** 1 Division of Occupational and Environmental Medicine, UConn Health, Farmington, Connecticut, United States of America; 2 Department of Epidemiology, Harvard T.H. Chan School of Public Health, Boston, Massachusetts, United States of America; 3 Beth Israel Deaconess Medical Center Cardiovascular Epidemiology Research Unit, Harvard Medical School, Boston, Massachusetts, United States of America; 4 Department of Environmental Health, Harvard T.H. Chan School of Public Health, Boston, Massachusetts, United States of America; 5 Department of Medicine, Massachusetts General Hospital/Harvard Medical School, Boston, Massachusetts, United States of America; School of Public Health of University of São Paulo, BRAZIL

## Abstract

**Purpose:** Exposure to secondhand smoke (SHS) has been associated with decreased heart rate variability (HRV). However, the time course of this association is unclear. Therefore, the objective of this study was to investigate the association between 15–240 minute SHS-related fine particulate matter (PM_2.5_) moving averages and indices of HRV. **Methods:** With a panel study design, we used personal monitors to continuously measure PM_2.5_ and HRV of 35 participants who were exposed to SHS for approximately 6 hours. **Results:** We observed negative, significant associations between 5-minute HRV indices and 15 minute PM_2.5_ moving averages and 240 minute PM_2.5_ moving averages: there was a significant (p<0.01) 7.5% decrease in the 5-minute square root of the mean squared differences of successive normal heart beats associated with (RMSSD), and a significant (p<0.01) 14.7% decrease in the 5-minute high frequency (HF) power associated with the 15 minute PM_2.5_ moving averages; there was also a significant (p<0.01) 46.9% decrease in the 5-minute RMSSD, and a significant (p<0.01) 77.7% decrease in the 5-minute high frequency (HF) power associated with the 240 minute PM_2.5_ moving averages. **Conclusions:** Our findings that exposure to SHS related PM_2.5_ was associated with HRV support the hypothesis that SHS can affect the cardiovascular system. The negative associations reported between short and longer term PM_2.5_ and HRV indicate adverse effects of SHS on the cardiovascular system.

## Introduction

Secondhand smoke (SHS) is associated with adverse chronic cardiovascular outcomes such as coronary heart disease[1, 2]. However, the mechanism by which SHS affects cardiovascular outcomes is still being investigated. SHS is believed to cause acute adverse physiological responses that, when repeated, may lead to damage to the cardiovascular system that can accumulate and lead to chronic conditions over time[3].

SHS, which is composed of fine particulate matter (PM_2.5_), may acutely affect the autonomic nervous system. PM_2.5_ has the ability to travel deep into the alveolar regions of the lungs where it can exert local and systemic pulmonary and cardiovascular health effects[4]. Several studies have demonstrated that participants had decreased heart rate variability (HRV) after exposure to SHS[5–7]. HRV, the beat-to-beat variability of the R-R interval of successive normal beats on an electrocardiogram (ECG), reflects the balance of sympathetic and parasympathetic autonomic nervous system activation[8]. HRV can be quantified using different measures including the standard deviation of the interval between normal heart beats (SDNN), the square root of the mean squared differences of successive normal heart beats (RMSSD), the power in the low and high frequency range of the heart rate signal (LF and HF power), and the LF/HF ratio[9]. Decreased HRV, as indicated by decreased SDNN, RMSSD, LF power, and HF power and an increased LF/HF ratio, indicates that there is imbalance in the autonomic nervous system, with increased sympathetic or decreased parasympathetic activation. This imbalance may lead to a release of hormones and inflammatory cytokines that can cause endothelial dysfunction, decreased vasodilation, and, if repeated chronically, increased risk for cardiovascular disease[10].

While previous studies have reported effects of SHS on HRV after exposures of differing durations, the time course of the autonomic response to SHS has not been investigated systematically. Studies of the effect of air pollution on HRV have indicated that there may be both short-acting (minutes) and longer-acting (hours) components to the mechanism of the effect of particulate matter on HRV (e.g. [11]). Information on the time course of the effects SHS on HRV would provide further insight into the mechanism of this relationship as well as inform exposure assessment strategies for future studies.

The objective of this study was to investigate the time course of the association between SHS-related PM_2.5_ exposure and HRV. In this study, participants were exposed to PM_2.5_ from SHS for approximately 6 hours. We investigated a series of PM_2.5_ moving averages ranging from 15 minutes to 240 minutes. We expected that higher short and long term PM_2.5_ moving averages would both be associated with lower HRV as indicated by lower 5-minute SDNN, RMSSD, LF power, and HF power and increased LF/HF ratio values.

## Methods

### Study Characteristics

Boilermaker construction workers were recruited to participate in this repeated-measures panel study, which was part of a larger study investigating welding and health effects [12, 13]. Eligible participants were members of a local boilermaker union in Quincy, Massachusetts, USA who were currently non-smokers. Participants were invited to participate in the study through letters sent by union leadership. The participants in the current study included 35 non-smoking workers and retirees, recruited over four site visits in June 2010, January and June 2011, and June 2012. Workers were allowed to participate during each of the four sampling periods. Seven workers participated in the study at two sampling periods, and two participated at three sampling periods. None of the workers had welded for at least three days prior to their measurement.

On the day of the measurement, participants stayed in an enclosed welding training room along with smoking welders, who were allowed to smoke during the measurements and therefore provided the SHS exposure sources. Participants were measured for approximately 6 hours when they performed book work, read, played card games, and completed questionnaires. All measurements were made in the same location under the same set up. All measurements began at about 7am to 9am in the morning and ran through the mid-afternoon. The study was approved by the Harvard School of Public Health Institutional Review Board and written informed consent was obtained from all participants.

### Secondhand Smoke PM_2.5_ Exposure Assessment

Participants wore Sidepak^™^ Aerosol Monitors (TSI, Inc., St. Paul, MN) during the measurements to obtain minute-to-minute average, personal breathing zone PM_2.5_ concentrations, which were used to quantify each participant’s individual SHS exposures[7, 14]. 15, 30, 60, 120, 180, and 240 minute PM_2.5_ moving averages were calculated from the minute-to-minute averages. We expected that the SHS was the primary and predominant source of PM_2.5_ in the measurement room.

### Heart Rate Variability

Participants wore standard ambulatory electrocardiogram (ECG) Holter monitors during the measurements. Some participants continued to wear Holter monitors after the measurement until the next morning. Holter monitors continuously and non-invasively record electrical signals from the heart that can be used to assess heart rate variability. The ECG records were analyzed by trained technicians, blind to exposures, in the Cardiovascular Epidemiology Research Unit of Beth Israel Deaconess Medical Center, and the HRV parameters of SDNN, RMSSD, LF power, HF power, and the LF/HF ratio were calculated. The continuous HRV data were summarized in 5 minute intervals throughout the measurement period.

### Statistical Analysis

The PM_2.5_ moving average and HRV measurements were aligned using the time stamps attached to each data stream. Separately for each 15 minute to 240 minute PM_2.5_ moving average, we used linear mixed regression models with the moving averages as the independent variables and HRV from the 5 minute interval at the end of the moving average (5-minute HRV indices) as the dependent variables (participant as random effect). An autoregressive covariance structure was assumed. The HRV data were natural-log transformed to ensure normality. All models were adjusted for each participants’ age and body mass index (treated continuously), and time of day (time varying, 0:00–6:00/6:00–12:00/12:00–18:00/18:00–24:00). Because statin use is known to blunt the cardiovascular response (Schwartz et al. 2005), we performed a sensitivity analysis excluding participants who reported using statin medications. We also performed a sensitivity analysis with only participants with cardiovascular and health risk factors such as diabetes, asthma, emphysema, chronic bronchitis, high blood pressure, metabolic syndrome, or history of prior cardiovascular event. Finally, we performed a sensitivity analysis removing any repeated measurements from the same participant. We considered two-sided p<0.05 to be significant.

## Results

The study population consisted of 35 men. Seven of these men participated in the study at two sampling periods, and two participated at three sampling periods, for a total of 46 observations. Descriptive statistics for participants are summarized in Table 1 and for exposure are summarized in Table 2.

**Table 1:** Participant demographics.

	N (%)	Mean (range)
Participants, n	35 (100)	
Statin Use	5 (14)	
Cardiovascular and health risk factors^1^	10 (29)	
Age, years		44 (21–71)
Body mass index, (kg/m^2^)		27 (18–37)

^1^ Including diabetes, asthma, emphysema, chronic bronchitis, high blood pressure, metabolic syndrome, or history of prior cardiovascular event

**Table 2:** Exposure characteristics for overall PM_2.5_ exposure and the 15–240 minute PM_2.5_ moving averages.

	Arithmetic Mean	Standard Deviation	5th Percentile	25th Percentile	50th Percentile	75th Percentile	95th Percentile
Overall	0.17	0.39	0.01	0.01	0.04	0.16	0.72
15-min	0.18	0.30	0.01	0.02	0.05	0.19	0.81
30-min	0.18	0.28	0.01	0.02	0.07	0.21	0.81
60-min	0.19	0.26	0.01	0.03	0.08	0.26	0.77
120-min	0.21	0.24	0.01	0.05	0.10	0.33	0.74
180-min	0.23	0.22	0.02	0.06	0.12	0.37	0.73
240-min	0.24	0.21	0.02	0.06	0.20	0.36	0.65

Note: all units are mg/m^3^

Among our entire population, we observed significantly lower 5-minute SDNN, RMSSD, and HF power indices and significantly higher 5-minute LF/HF ratio indices associated with the 15 minute PM_2.5_ moving averages (Table 3). For example, the 15 minute PM_2.5_ moving average was associated with 7.5% lower 5-minute square root of the mean squared differences of successive normal heart beats (RMSSD) (p<0.01) and 14.7% lower 5-minute high frequency (HF) (p<0.01). We also observed significantly lower 5-minute RMSSD associated with the 30 minute PM_2.5_ moving averages. We observed significantly lower 5-minute HRV indices associated with the 240 minute PM_2.5_ moving average. For example, the 240 minute PM_2.5_ moving average was associated with 46.9% lower 5-minute RMSSD (p<0.01) and 77.7% lower 5-minute high frequency (HF) power. We observed significant (p<0.01), positive associations between 120 minute PM_2.5_ moving averages and 5-minute SDNN, RMSSD, LF power, and HF power indices. When we removed participants reporting statin use, we observed similar results as observed in the entire population: we observed significantly lower HRV associated with the 15 minute and 240 minute PM_2.5_ moving averages, and significantly higher 5-minute HRV indices associated with the 120 minute PM_2.5_ moving averages. The effect sizes were slightly larger when participants with statins were removed. Among participants with cardiovascular and health risk factors, we observed significantly lower 5-minute SDNN, RMSSD, and HF power indices associated with the 30 minute and 60 minute PM_2.5_ moving averages and significantly higher 5-minute LF/HF ratio associated with the 120 minute PM_2.5_ moving averages. Sensitivity analyses still demonstrated fluctuations between negative and positive associations between PM_2.5_ and HRV among participants not taking statins, those with cardiovascular and health risk factors, and after removing any repeated measurements from the same participants measured on different days.

**Table 3:** Beta coefficients, standard errors, and p-values for the effect of 15–240 minute PM2.5 moving average on 5-min ln(HRV) parameters among our entire population (n = 35).

	SDNN			RMSSD			LF Power			HF Power			LF/HF Ratio		
	Beta-coefficient^1^,^2^,^3^	Standard Error	p-value	Beta-coefficient^1^,^2^,^3^	Standard Error	p-value	Beta-coefficient^1^,^2^,^3^	Standard Error	p-value	Beta-coefficient^1^,^2^,^3^	Standard Error	p-value	Beta-coefficient^1^,^2^,^3^	Standard Error	p-value
15 min	-0.0676	0.0259	**<0.01**	-0.0782	0.0233	**<0.01**	0.0694	0.0471	0.14	-0.1593	-0.1593	**<0.01**	0.2321	0.0432	**<0.01**
30 min	-0.0458	0.0283	0.11	-0.0633	0.0256	**0.01**	0.1154	0.0515	**0.03**	-0.1145	0.0595	0.05	0.2338	0.0474	**<0.01**
60 min	-0.0150	0.0326	0.65	0.0005	0.0297	0.87	0.1804	0.0598	**<0.01**	0.0278	0.0687	0.6858	0.1583	0.0549	**<0.01**
120 min	0.1641	0.0479	**<0.01**	0.2455	0.0449	**<0.01**	0.3610	0.0899	**<0.01**	0.4535	0.1043	**<0.01**	-0.0732	0.0812	0.37
180 min	0.1317	0.0873	0.13	0.0431	0.0854	0.61	0.1031	0.0086	0.53	-0.1630	0.1959	0.41	0.2899	0.1420	**0.04**
240 min	-0.1026	0.1635	0.53	-0.6339	0.1671	**<0.01**	-0.4139	0.3085	0.18	-1.5007	0.3801	**<0.01**	0.9132	0.2539	**<0.01**

^**1**^ **Adjusted for** age, body mass index, and time of day

^2^ A one-unit change in PM_2.5_ moving average corresponds to change in the ln(HRV) parameter by the amount of the beta-coefficient.

^3^ Percentage change can be calculated from the beta coefficients as: percentage(%) = [exp(beta coefficients)-1]*100.

## Discussion

The objective of this study was to investigate the time course of the association between SHS related PM_2.5_ exposure and HRV. We expected that increased short and longer term PM_2.5_ moving averages of SHS exposure would both be associated with decreased HRV as indicated by lower 5-minute SDNN, RMSSD, LF power, and HF power and increased LF/HF ratio indices. Among our whole population, we observed associations between PM_2.5_ moving average exposure and both increases and decreases in HRV, with short term (15 minute) and longer tern (240 minute) PM_2.5_ moving averages being associated with decreased HRV while 120 minute moving average PM_2.5_ levels were associated with increased HRV.

Our observation that increased SHS related PM_2.5_ exposure was associated with decreased HRV is line with previous studies of the effect of SHS on HRV. Pope et al. observed decreases in SDNN when participants were exposed to SHS for two hours compared to when participants were unexposed[5]. Wilson et al. observed significant decreases in SDNN and RMSSD post-shift compared to pre-shift among non-smoking bar and restaurant workers in establishments where smoking was permitted[6]. Zhang et al. observed significant decreases in SDNN, RMSSD, LF power, and HF power immediately after six hours of SHS exposure[7]. Felber Dietrich et al. reported that participants chronically exposed to SHS at work or home had decreased LF/HF ratios compared to participants not exposed to SHS[15]. Finally, Rajkumar et al. estimated that participants experienced increases in RMSSD and HF power 3–12 months after a smoking ban[16].

Our observations of decreased HRV associated with increased short and longer term PM_2.5_ moving averages correspond to the results of previous studies of the effect of air pollution on HRV. Magari et al. observed decreases in participants’ SDNN corresponding to increased 15-minute and 4-hour moving workday PM_2.5_ moving averages[11]. Magari et al. observed decreases in SDNN associated with air pollution related PM_2.5_ moving averages, with the strongest effects at 3-hour moving averages[17]. He et al. observed significant decreases in SDNN, HF power, and LF power associated with increases in 1–6 hour air pollution related PM_2.5_ moving averages[18]. Huang et al. observed decreases in participants’ SDNN and RMSSD corresponding to increased 1-hour to 4-hour household PM_2.5_ moving averages[19].

Our observation of increased HRV associated with increased PM_2.5_ exposure corresponds to the results of some previous studies. While there seems to be a consensus that increased PM_2.5_ exposure is associated with decreased HRV, and therefore increased risk of adverse cardiovascular outcomes, among older and more susceptible populations, the results are not entirely consistent for younger, healthier populations[10]. For example, Shields et al. observed increases in SDNN, HF, and LF and decreases in the LF/HF ratio associated with moving 5–90 minute traffic related PM_2.5_ moving averages among a young (mean age = 35) population of researchers[20]. Wu et al. observed overall decreases in HRV parameters associated with 5–240 minute PM_2.5_ moving averages, but also observed heterogeneity among responses with some participants having positive associations[21]. One difference between the results of our study and these previous studies is that the previous studies did not observe both significant positive and negative associations between PM_2.5_ and HRV, while we did observe negative associations for our shortest (15 minute) and longest (240 minute) PM_2.5_ moving averages and positive associations in between (120 minute moving averages). The PM_2.5_ concentrations observed in our study were somewhat higher in these previous studies and came from a different source (SHS), which may have affected the HRV response. For example, Shields et al. observed PM_2.5_ concentrations of approximately 3 mg/m^3^ from diesel exhaust sources, while Wu et al. observed PM_2.5_ concentrations of less than 1 mg/m^3^ from particulate air pollution. Perhaps in our study we observed some adaption to maintain homeostasis, that was then overwhelmed after longer durations of higher intensity SHS exposure[22]. Our sensitivity analyses still demonstrated fluctuations between negative and positive associations between PM_2.5_ and HRV among participants not taking statins, those with cardiovascular and health risk factors, and after removing any repeated measurements from the same participants measured on different days, providing no evidence of any sub-samples of participants that may have had different responses from our overall findings. In addition, we performed some other analyses to investigate more closely whether these findings could have been due to diurnal or circadian variations by changing our assumptions about how the “time of day” variable was modelled and how long each participant was measured, and also removing one outlier residual, but still observed the same trends. Future studies should investigate whether our findings of fluctuations between positive and negative associations between SHS and HRV can be replicated or if perhaps this just represents noise in the data.

The results of this study must be viewed in light of the study’s limitations. With only 35 participants in total, we had a relatively small sample size, especially for the sensitivity analyses. However, the repeated-measures of HRV and PM_2.5_ helped to increase power to detect associations as well as to eliminate confounding. Our study population consisted entirely of males, and was relatively young and healthy. Therefore, our results may not be generalizable to other populations. We did not measure respiratory rate or activity level during the measurement periods, which are known to affect HRV. The Sidepak^™^ Monitors are unable to distinguish between types of PM_2.5_, but simply provide a measure of the overall PM_2.5_ intensity. We expect that SHS was the primary and predominant source of PM_2.5_ exposure within our measurement area, but it is possible that other sources may have introduced some additional PM_2.5_ into the room during our measurements. We also were unable to test for associations between components of SHS other than PM_2.5_ and HRV, although SHS is a mixture of many factors that may have differing effects on HRV. Regardless of these limitations, this was the first study to demonstrate the time course of the HRV response to SHS exposure, providing further support for the hypothesis that SHS affects autonomic functioning and insight into the mechanism of the relationship between SHS and adverse cardiovascular outcomes.

In conclusion, this study demonstrated a relationship between PM_2.5_ moving averages of different lengths and HRV. Our findings that exposure to SHS related PM_2.5_ was associated with HRV support the hypothesis that SHS can affect the cardiovascular system. The negative associations reported between short and longer term PM_2.5_ and HRV indicate adverse effects of SHS on the cardiovascular system, and support arguments for the reduction and elimination of SHS from the environment.

## Supporting Information

S1 File Minimal dataset to replicate the study’s underlying findings. (XLSX)
